# Pregabalin Add-On vs. Dose Increase in Levetiracetam Add-On Treatment: A Real-Life Trial in Dogs With Drug-Resistant Epilepsy

**DOI:** 10.3389/fvets.2022.910038

**Published:** 2022-07-06

**Authors:** Sandra R. P. Kriechbaumer, Konrad Jurina, Franziska Wielaender, Henning C. Schenk, Tanja A. Steinberg, Sven Reese, Gesine Buhmann, Stefanie Doerfelt, Heidrun Potschka, Andrea Fischer

**Affiliations:** ^1^Centre for Clinical Veterinary Medicine, Clinic of Small Animal Medicine, Ludwig-Maximilians-University Munich, Munich, Germany; ^2^AniCura Small Animal Clinic Haar, Haar, Germany; ^3^Small Animal Clinic Lüneburg, Lüneburg, Germany; ^4^Department of Veterinary Sciences, Institute of Anatomy, Histology and Embryology, Ludwig-Maximilians-University Munich, Munich, Germany; ^5^Department of Veterinary Sciences, Institute of Pharmacology, Toxicology, and Pharmacy, Ludwig-Maximilians-University Munich, Munich, Germany

**Keywords:** epilepsy, seizures, dog, drug-resistance, pregabalin, levetiracetam, drug level

## Abstract

Epilepsy is a common neurological disorder affecting 0.6–0.75% of dogs in veterinary practice. Treatment is frequently complicated by the occurrence of drug-resistant epilepsy and cluster seizures in dogs with idiopathic epilepsy. Only few studies are available to guide treatment choices beyond licensed veterinary drugs. The aim of the study was to compare antiseizure efficacy and tolerability of two add-on treatment strategies in dogs with drug-resistant idiopathic epilepsy. The study design was a prospective, open-label, non-blinded, comparative treatment trial. Treatment success was defined as a 3-fold extension of the longest baseline interseizure interval and to a minimum of 3 months. To avoid prolonged adherence to a presumably ineffective treatment strategy, dog owners could leave the study after the third day with generalized seizures if the interseizure interval failed to show a relevant increase. Twenty-six dogs (mean age 5.5 years, mean seizure frequency 4/month) with drug-resistant idiopathic epilepsy and a history of cluster seizures were included. Dogs received either add-on treatment with pregabalin (PGB) 4 mg/kg twice daily (14 dogs) or a dose increase in levetiracetam (LEV) add-on treatment (12 dogs). Thirteen dogs in the PGB group had drug levels within the therapeutic range for humans. Two dogs in the PGB group (14.3%; 2/14) and one dog in the LEV group (8.3%; 1/12) achieved treatment success with long seizure-free intervals from 122 to 219 days but then relapsed to their early seizure frequency 10 months after the study inclusion. The overall low success rates with both treatment strategies likely reflect a real-life situation in canine drug-resistant idiopathic epilepsy in everyday veterinary practice. These results delineate the need for research on better pharmacologic and non-pharmacologic treatment strategies in dogs with drug-resistant epilepsy.

## Introduction

Epilepsy is a common neurologicaldisorder affecting 0.6–0.75% of dogs in veterinary practice (1, 2). Treatment is complicated by the frequent occurrence of drug-resistant epilepsy in dogs with idiopathic epilepsy (3, 4).

In clinical practice in Europe, legal regulations define the use of phenobarbital (PB), potassium bromide (KBr), and imepitoin as first-line antiseizure medications (ASMs) in dogs with idiopathic epilepsy. Imepitoin is only licensed for the treatment of single seizures. Its efficacy for cluster seizures is a matter of ongoing debate (5–8). Dog owners and veterinarians considered the development of new ASMs among the three most important research topics for the future (9). Evidence for the efficacy of non-licensed ASMs in dogs is poor as there are only few prospective controlled studies evaluating treatment strategies in dogs with drug-resistant epilepsy (10–13). Considering that applying a placebo to individuals with drug-resistant epilepsy may be unethical, there is a trend toward comparative active-controlled studies in epilepsy research (4, 14–20).

We, therefore, designed a prospective clinical trial comparing two treatment strategies for dogs with drug-resistant idiopathic epilepsy. The first treatment strategy was pregabalin (PGB) add-on therapy with 4 mg/kg q12 h PO (BID). The second treatment strategy was a 30% increase in the dose of levetiracetam (LEV) add-on treatment given q8 hours (TID). Both LEV and PGB target non-GABAergic pathways and therefore likely exhibit additive antiseizure efficacy to first-line GABAergic drugs (5, 17). PGB showed promising antiseizure efficacy TID dosing in a previous uncontrolled pilot study (21) and had a favorable pharmacokinetic profile suggesting that even BID dosing may be sufficient to achieve effective serum concentrations (22). The American College of Veterinary Internal Medicine (ACVIM) consensus statement on seizure management in dogs recommended LEV as an add-on medication based on evidence and risk profile (23). Both ACVIM and International Veterinary Epilepsy Task Force (IVETF) recommendations suggested an increase in dosage or more frequent application of LEV add-on treatment to overcome tolerance issues (5, 23–25). Little is known about the efficacy of these two treatment strategies in canine drug-resistant idiopathic epilepsy in real life.

Our clinical trial aimed to evaluate and compare these two treatment strategies in dogs with drug-resistant epilepsy. Furthermore, we hypothesized that add-on treatment with 4 mg/kg PGB given BID to dogs would achieve serum concentrations within the therapeutic range for humans.

## Materials and Methods

The study design was a prospective, open-label, non-blinded, comparative treatment trial for the evaluation of two add-on treatment strategies.

Dogs with drug-resistant idiopathic epilepsy and monthly generalized tonic–clonic (GTC) seizures (interseizure intervals ≤ 40 days) were recruited at study centers and with study calls placed on websites. Idiopathic epilepsy was diagnosed based on a history of epileptic seizure onset between 6 months and 6 years, review of video footage, documentation of unremarkable physical and neurologic examination, unremarkable blood tests (hematology, serum biochemistry), and bile acid stimulation test. Brain imaging with magnetic resonance imaging (MRI) and cerebrospinal fluid (CSF) analysis (tier II) was encouraged but not mandatory (26). Only dogs with drug-resistant idiopathic epilepsy as defined previously were included (4), i.e., failure to achieve seizure freedom with ≥2 ASMs. This required documentation of serum concentrations within the therapeutic range (PB > 20 mg/L, KBr > 1,000 mg/L) and/or treatment with adequate dosages (imepitoin 20–30 mg/kg BID; LEV, 15–20 mg/kg TID) unless these were not tolerated. Study participation was denied for the following criteria: dogs younger than 1 year or older than 12 years of age, dogs with post-traumatic epilepsy, ASMs or their dosages modified in the 2 months preceding patient enrolment, dogs treated with immunosuppressants or anti-inflammatory drugs, or in case of any other concurrent relevant metabolic, endocrine, neoplastic, immune, or cardiac disease. Four months (112 days) were selected as the baseline period to provide information on a minimum of three seizure cycles in dogs with monthly seizures. Baseline seizure data were collected from an online questionnaire and written seizure logs provided by the dog owners. Only GTC seizures with and without focal onset were considered. Cluster seizures and status epilepticus were identified as previously described (27). The following baseline parameters were calculated: longest interseizure interval during baseline (T1), and monthly seizure frequency (MSF) during baseline. For T1, the longest seizure-free period between two GTC seizures during baseline was extracted from written seizure logs. For MSF, GTC seizure counts during the 112 days baseline period were divided by four (28 days/month), and each GTC seizure of a cluster event was counted. Furthermore, monthly seizure day frequency (MSDF) and cluster day frequency (MCDF) were calculated to compare baseline characteristics between treatment groups. Owners provided details regarding seizure onset and semiology, triggers, prodromal signs, duration of seizures, postictal signs, and suspicious focal seizure signs. Owners were asked to assign numerical scores for seizure severity (score 1–5; 1 mild, 5 severe), quality of life of their dogs (0–10; 0, poor; 10, excellent), behavioral changes (playfulness, activity level, 0–10; 0, very low; 10, excellent), and observations on possible side effects of ASMs (weakness, ataxia, disorientation, sedation, restlessness, and increased appetite; 0–10; 0, none; 10, severe). Owners were also asked to provide home videos if severe side effects were reported.

### Treatment

Dogs were assigned to either PGB add-on treatment (PGB group) or treatment with increased dosages of LEV add-on treatment (LEV group, only for dogs already treated with LEV). Allocation to treatment groups followed a stratified randomization approach. Matching criteria aimed to establish pairs of dogs with similar disease severity and characteristics (Table 1). Dogs were randomized to treatment groups (https://www.random.org) if there was no matching partner available. Dogs in the PGB group received add-on treatment with PGB 4 mg/kg BID. PGB was supplied in different tablet sizes, e.g., 100, 75, 50, and 25 mg, which could be divided into halves (PregaTab^®^, Neuraxpharm, Germany). In order to avoid excessive sedation, the starting dose was 1 mg/kg BID, the dosage was increased every 4th day by 1 mg/kg BID until the target dose of 4 mg/kg BID was reached resulting in a 2-week titration phase. For dogs in the LEV group, the baseline dosage of LEV add-on treatment given TID was increased by 30% without a change in brand or manufacturer, and a 1-week titration phase was considered. There was no change in emergency treatment protocols. Dog owners were able to leave the study after the 3rd GTC seizure day during the treatment phase (individual endpoint) if there was no relevant short-term effect, which was defined as ≥1.5-fold extension of the longest interseizure interval of the 4-month baseline period (T1). Seizures during the titration phase were not counted. Study exit was also offered if status epilepticus or severe side effects occurred.

**Table 1:** Matchingcriteria.

**Prioritization**	**Definition of criteria**	**Subgroup**
C1	Longest interval between two seizure days during 4 months baseline period (T1)	S1: ≤ 14 days S2: > 14 days
C2	Monthly seizure day frequency (MSDF)	S1: > 2 S2: ≤ 2
C3	Cluster seizures	S1: yes S2: no
C4	Age at onset of epilepsy	S1: ≤ 2 years of age S2: > 2 years of age
C5	Predisposed breed	S1: yes S2: no

*Matching criteria were defined according to potential impact on study outcome with C1 being most and C5 being least influencing. Matched pair partners had to agree in ≥3 criteria subgroups in ascending order. C, criterion; S, subgroup*.

### Voluntary Study Extension

Owners were offered to extend study participation beyond the individual endpoint (3rd GTC seizure day) and remain in the study for up to 6 months or even longer. In case of treatment failure, owners in the PGB group were offered to increase the daily PGB dose and apply PGB add-on treatment TID. Likewise, in the LEV group, owners were offered to increase the dose of LEV TID add-on treatment by another 30%.

### Evaluation of Efficacy

Only GTC seizures and the longest interseizure interval during the treatment phase (longest ISI) were considered. Treatment success was defined as seizure freedom or three-fold extension of the longest baseline interseizure interval (longest ISI ≥ 3 T1; minimum 3 months) (4, 5, 28). Furthermore, time (days) to the 3rd seizure day in the treatment phase was calculated as an additional outcome parameter for all dogs (14, 29–32). For the dogs, which remained in the study for ≥56 days, mean MSF was calculated for the first 56 days of the treatment phase and compared to the 4-month baseline period. Drug retention rates 6 months after treatment initiation and long-term follow-up data were obtained from all dogs.

### Statistical Analysis

The sample size was calculated taking an alpha error probability of *p* < 0.05, a power > 80%, and a postulated large effect (Cohen's *d* 0.8) as a basis. Power-based sample size calculation resulted in a minimum of 12 dogs in each treatment arm using a stratified matched pair design. The generalized mixed linear model was used to compare the PGB group to the LEV group. The matched pairs were defined as subjects and the grouping variable as the repeated measure and fixed effect. The individual dogs were added as a random effect. Depending on the kind of target variable, the target distribution was defined as normal distributed (tested by visualization of Q–Q plots and the Kolmogorov–Smirnov test), gamma distributed with log link, or binomial. In the case of a multinomial target variable (scores), we used the generalized linear model without considering the random effect of the individual dogs. The reason is the technical restrictions of SPSS in the case of multinomial data. Correlations between drug serum concentrations and extension of the interseizure interval were assessed for both treatment groups by calculating the correlation coefficient Spearman's rho. Time to the 3rd GTC seizure day was evaluated with Kaplan–Meier curves and log-rank test (bivariate) and Cox regression analysis in a multivariate setting. Changes in MSF compared to baseline were only assessed for dogs with ≥56 days of study participation. The analysis was performed by using the statistical software package SPSS 28.0.1.0 (IBM, Ehningen, Germany).

## Results

### Study Population

In total, 142 dogs were screened for eligibility and 26 dogs (18 male, eight female; mean age 5.5 years, range 1.9–11.3) with idiopathic epilepsy were enrolled in the study (Table 2; Supplementary Table S1). Breeds were as follows: mixed breed (10 dogs), Australian Shepherd (two dogs), Golden Retriever (two dogs), Beagle, Border Terrier, Boxer, Cane Corso Italiano, Elo, Labrador Retriever, Magyar Vizsla, Old German Shepherd, Old English Bulldog, Rhodesian Ridgeback (wild-type JME gene variant *DIRAS1*), Siberian Husky, and White German Shepherd (one dog each). Age at seizure onset was 2.3 years (mean; range 8 months−4.8 years). All dogs experienced generalized seizures with or without a focal onset; all dogs had a history of cluster seizures (24 dogs during a 4-month baseline period, 2 dogs before baseline). The focal onset of generalized seizures was suspected in 46.2% of the dogs (12/26), and this was the predominant seizure type in 26.9% of the dogs (7/26). Additional episodes suspicious for focal epileptic seizures (e.g., twitches and jerks) occurred in 61.5% (16/26) of dogs. Diagnosis of idiopathic epilepsy was supported by unremarkable brain imaging and CSF analysis in 11 dogs (eight MRI, two CT) and unremarkable brain imaging only in three more dogs (two MRI, one CT). EEG was performed on three dogs. Dogs in the PGB and LEV groups did not differ regarding seizure frequency, seizure day frequency, cluster seizures, drug serum concentrations (PB and KBr), and other parameters (*p* < 0.05; Table 2). All dogs had minimum two failed adequate ASM trials. At study inclusion, 13 dogs were treated with two ASMs, seven dogs with three ASMs, four dogs with four ASMs, and two dogs with one ASM. Detailed information on the current and previous ASMs is provided in Supplementary Table S1 for each dog. All dogs were treated with PB and KBr at study inclusion or before, except four dogs (one PGB, three LEV), which had yet not received KBr due to lack of drug supply in 2020/2021. Thirteen dogs were previously treated with imepitoin, but treatment was discontinued due to lack of efficacy in twelve dogs and side effects in one dog. Thirteen dogs had yet not received imepitoin because of a history of cluster seizures.

**Table 2:** Baseline characteristics of enrolled dogs with drug-resistant idiopathic epilepsy.

**Parameter**	**All dogs**	**Pregabalin**	**Levetiracetam**	***p*-value**
	***n* = 26**	***n* = 14**	***n* = 12**	
Predisposed breed	15 dogs (58%)	9 dogs (64%)	6 dogs (50%)	0.805
Body weight, mean (range)	29.5 kg (9–50)	29.2 kg (9–50)	29.8 kg (20–38)	0.932
Sex	18 males (69%)	9 male (64%)	9 male (75%)	1.000
Age at onset of IE	2.3 y (0.7–4.8)	2.2 y (0.7–4.8)	2.5 y (0.9–4.6)	0.599
Age at study inclusion	5.5 y (1.9 −11.3)	4.8 y (1.9–9.6)	6.1 y (4.0–11.3)	0.367
GTC seizures	26 (100%)	14 (100%)	12 (100%)	1.000
- frequent focal onset	5 (19.2%)	2 (14.3%)	3 (25.0%)	1.000
- rare focal onset	7 (26.9%)	4 (28.6%)	3 (25.0%)	1.000
Susp. focal seizure signs	16 (61.5%)	8 (57.1%)	8 (66.7%)	1.000
T1, mean (range)	26.0 d (6–39)	26.4 d (10–38)	25.5 d (6–39)	0.847
MSF, mean (range)	4.0 (1.3–9.8)	4.0 (1.3–9.8)	3.9 (1.5–9.5)	0.923
MSDF, mean (range)	2.6 (1.0–9.3)	2.6 (1.0–4.3)	2.5 (1.0–9.3)	0.872
MCDF, mean (range)	1.0 (0.3–3.5)	1.1 (0.3–3.5)	0.9 (0.0–2.3)	0.607
Duration of postictal signs, mean (range)	38 min (0.5–120)	42 min (5–90)	35 min (0.5–120)	0.746
Seizure severity score, mean (range 1–5)	3.7 (2–5)	3.8 (3–5)	3.6 (2–5)	0.713
Quality of life score, mean (range 1–10)	6.6 (2–10)	6.4 (2–10)	6.7 (3.5–9)	0.876
**Baseline treatment**
PB concentration, mean	26.6 mg/l	27.2 mg/l	25.9 mg/l	0.576
KBr concentration, mean	1,493.3 mg/l	1,503.8 mg/l	1,482.8 mg/l	0.950
No. ASMs, mean (range)	2.5 (1–3)	2.2 (1–3)	2.8 (1–3)	0.077
- phenobarbital	24 dogs	14 dogs	10 dogs	
- potassium bromide	17 dogs	11 dogs	6 dogs	
- levetiracetam	17 dogs	5 dogs	12 dogs	
- pregabalin	4 dogs	0 dogs	4 dogs	
- other (topiramate, gabapentin, amantadine)	3 dogs	1 dog	2 dogs	
Levetiracetam pulse therapy	15 dogs (57.7%)	8 dogs (57.1%)	7 dogs (58.3%)	1.000

*IE, idiopathic epilepsy; d, days; y, years; min, minutes; T1, longest interseizure interval during 4 months baseline period (days); MSF, monthly seizure frequency; MSDF, monthly seizure day frequency; MCDF, monthly cluster day frequency; GTC, generalized tonic–clonic; PB, phenobarbital; KBr, potassium bromide; No., number; ASM, antiseizure medication*.

In the LEV group, the mean dose of LEV at study inclusion was 23.8 mg/kg TID (range 17.2–37.8 mg/kg), which had been provided for a mean of 8.3 months. The mean baseline LEV serum concentration was 10.6 mg/L (0–27.6 mg/L, therapeutic range 10–40 mg/L; MVZ Labor Krone GmbH; Bad Salzuflen, Germany), whereas 50% of dogs had serum concentrations below the therapeutic range for humans. Behavioral changes and side effects of ASMs were commonly reported in both groups (Supplementary Table S2).

### Treatment Phase

**Figure 1:**
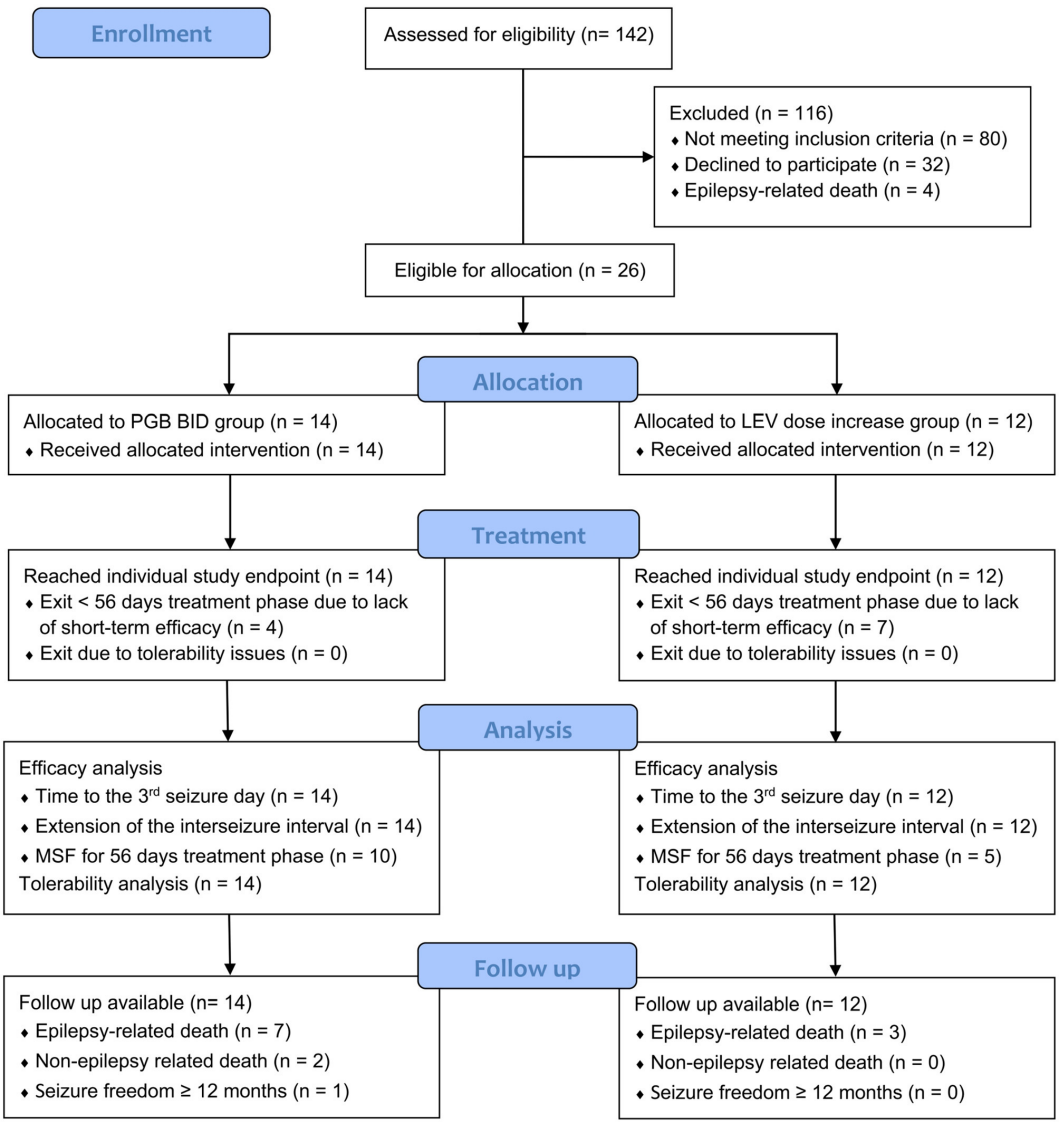
CONSORT flow diagram of study design. Twenty-six dogs were allocated to one of two treatment arms by stratified randomization. All dogs reached their individual study endpoint, which was minimum the 3rd day with generalized tonic–clonic seizures. Follow-up was available from all dogs. PGB, pregabalin; BID, dosing q12 hours; LEV, levetiracetam (33).

**Figure 2:**
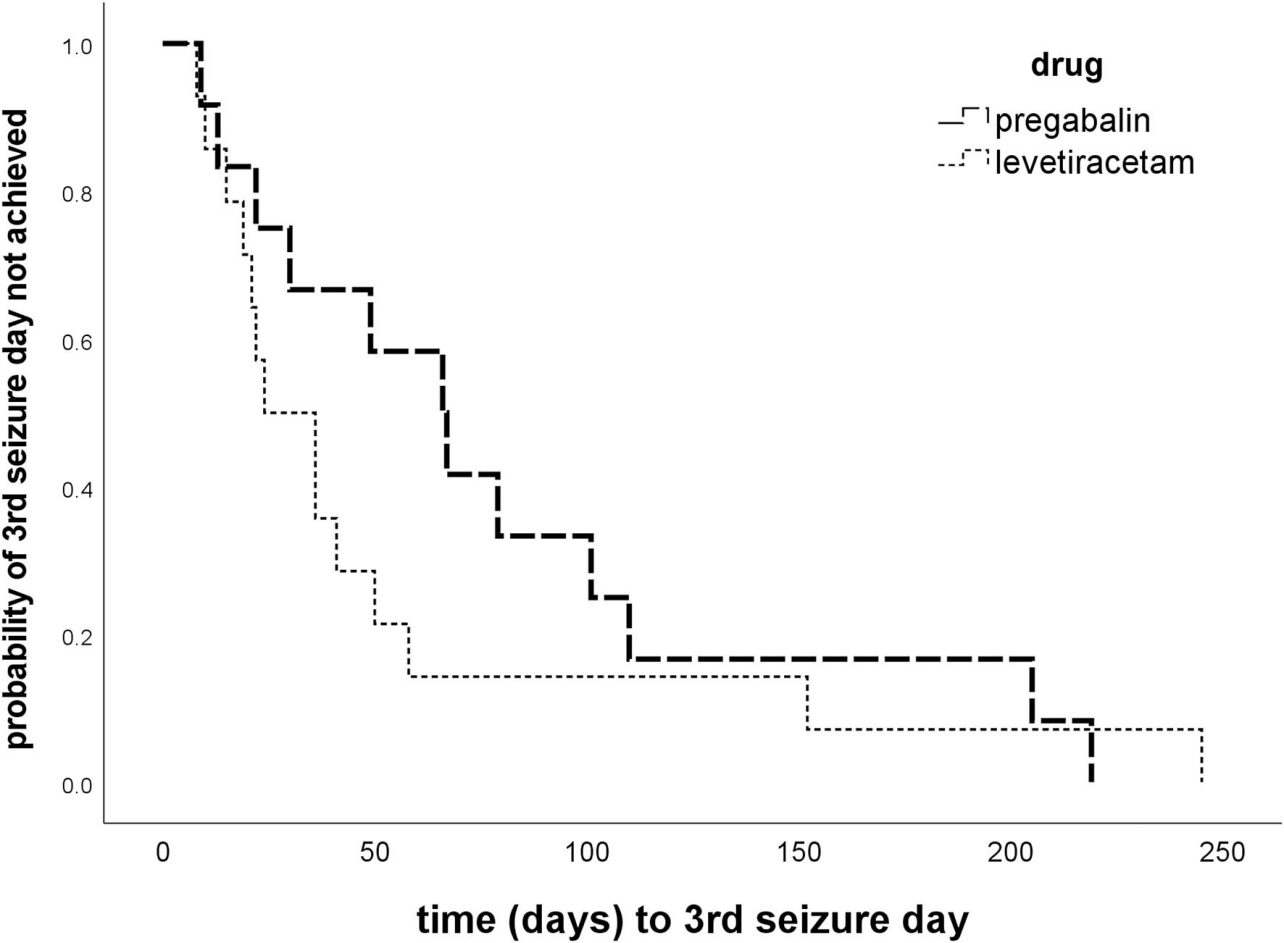
Time to 3rd GTC seizure day. Kaplan–Meier plots of time (days) to 3rd generalized tonic–clonic seizure day in the treatment phase show no difference between the pregabalin BID and levetiracetam dose increase group (*p* = 0.334).

All 26 dogs completed the titration phase and entered the treatment phase: 14 in the PGB group and 12 in the LEV group (Figure 1). Mean PGB dosage was 3.96 mg/kg BID (3.66–4.10 mg/kg). Dosage of LEV add-on treatment was increased from 30.4% (mean, range 25–34%) to 31.1 mg/kg TID (mean; range 23.0–50.3 mg/kg). All dogs were treated according to the study protocol until the 3rd day with generalized epileptic seizures or longer (Figure 2). The owners of 16 dogs (nine PGB and seven LEV) decided to extend the treatment phase beyond the 3rd GTC seizure day. Overall, dogs from the PGB group participated in the BID treatment phase of the study between 9 and 205 days (median 80; mean 97 days), and dogs from the LEV group between 22 and 245 days (median 49; mean 74 days).

### Efficacy

Overall success rates were low with no obvious difference in treatment success between the two treatment strategies (14.3 vs. 8.3%; *p* > 0.05). There was also no significant difference between the two treatment groups in time to the 3rd GTC seizure day (*p* = 0.334; Figure 2), duration of study participation, and proportions of dogs with ≥50% decrease in MSF in the first 56 treatment days (Table 3). The 3rd GTC seizure day occurred within 3 months in 77% of the dogs (20/26; 10 PGB, 10 LEV), between 3 and 6 months in 11.5% (3/26; 2 PGB, 1 LEV), and after 6 months of treatment in another 11.5% (3/26; 2 PGB, 1 LEV; Figure 2). The interseizure interval increased 1.61-fold (mean) in the PGB group and 1.49-fold in the LEV group (*p* = 0.681) compared to baseline (Table 3).

**Table 3:** Comparative evaluation of efficacy and tolerability of pregabalin add-on vs. dose increase in levetiracetam add-on treatment.

**All dogs (*n* = 26)**	**Pregabalin BID**	**Levetiracetam**	***p*-value**
	***n* = 14**	***n* = 12**	
Dogs with treatment success (longest ISI ≥ 3 T1)	2 (14.3%)	1 (8.3%)	1.000
Longest ISI (treatment phase), mean (median)	44.8 d (29.5)	36.7 d (15)	0.527
Extension of the interseizure interval, mean	1.61 T1	1.49 T1	0.681
Time to 3rd seizure day, mean (median)	75 d (62)	55 d (30)	0.500
Dogs exiting study <56 days of treatment	4 (28.6%)	7 (58.3%)	0.233
**Only dogs which participated** **≥56 days**	***n*** **=** **10**	***n*** **=** **5**	0.233
MSF 56 days treatment, mean (median)	2.4 (1.5)	2.6 (1.5)	0.841
MSF baseline, mean	3.4	5.5	0.145
% change in MSF, mean	−29.9%	−52.7%^*^	0.898
≥50% decrease in MSF	5 dogs (35.7%^#^)	2 dogs (16.7%^#^)	1.000
Cease of cluster seizures for ≥6 months	2 dogs (14.3%^#^)	1 dogs (8.3%^#^)	1.000
**Other parameters (all dogs)**	***n*** **=** **14**	***n*** **=** **12**	
Study participation, mean (median)	97 d (80)	74 d (49)	0.323
Duration of postictal signs, mean (range)	25 min (0.5–80)	22 min (10–45)	0.768
Seizure severity score, mean (range 1–5)	3.6	3.3	0.652
Suspicious focal seizure signs, no. dogs	6 (42.9%)	7 (58.3%)	0.515
Body weight at study completion, mean (range)	29.3 kg (9–48)	30.2 kg (19–38)	0.966
PB serum concentration, mean (% change)	25.9 mg/l (−4.8%)	24.9 mg/l (−3.9%)	0.721
KBr serum concentration, mean (% change)	1,541.1 mg/l (−2.4%)	1,873.2 mg/l (+26.3%)	0.414
Dogs with drug adaption because of side effects	5 (35.7%)	2 (16.7%)	1.000
Quality of life score, mean (range 0–10)	7.3 (2–10)	6.3 (0–9.5)	0.383
% change in quality of life score, mean	+12.8%	−6.3%	0.243

*Longest ISI, longest interseizure interval during treatment phase; T1, longest interseizure interval during 4 months baseline; d, days; y, years; min, minutes; MSF, monthly seizure frequency*.

* *The decrease of MSF in the levetiracetam group is biased by one dog with high cluster seizure burden during baseline and no cluster seizures during treatment phase*.

# *Intention-to-treat analysis, percentages were calculated considering all dogs within the group (14 pregabalin BID, 12 levetiracetam)*.

Pregabalin group (*n* = 14): Two dogs (14.3%; 2/14) achieved treatment success with a 3.3-fold and 6.4-fold extension of T1 corresponding to seizure-free intervals of 122 and 180 days (longest ISI). Time to the 3rd GTC seizure day was 205 and 219 days, respectively. Both dogs had GTC seizures with suspected focal onset (one rare motor, one frequent autonomic). In one of these dogs, bromide concentration increased from 1,075.0 mg/L at baseline to 1,779.0 mg/L at study completion, presumably after a change in diet due to a suspected allergic food reaction. All dogs but one achieved PGB serum concentrations within the human therapeutic range of the laboratory (2–5 mg/L; MVZ Labor Krone GmbH). Mean PGB serum concentration was 3.8 mg/L (range 1.4–7.8 mg/L; 14 dogs). There was a moderate positive correlation (rho = 0,515, *p* = 0.060) between PGB serum concentration and extension of the interseizure interval. Mean PGB serum concentration was 5.2 mg/L in the two dogs with treatment success compared to 3.6 mg/L (±1.76 SD; *p* = 0,144) in the other dogs. MSF, MSDF, and MCDF during baseline were negatively correlated with extension of the interseizure interval and time to the 3rd GTC seizure day (*p* < 0.05). Owners of four dogs (28.6%; 4/14) chose to leave the study before 56 days of treatment due to a perceived lack of short-term efficacy. Ten dogs adhered to the study protocol for ≥56 days. Of these, five dogs (5/14; 37.5%) experienced ≥50% decrease in MSF during the first 56 days of treatment when compared to baseline (4 months) but a decrease in MSF was only maintained in 21.4% (3 dogs) after 3 months and 14.3% (two dogs) after 6 months of treatment, respectively. The 6-month drug retention rate was 43% (6/14) in the PGB group.

Levetiracetam group (*n* = 12): One dog (8.3%) achieved treatment success with an 8.7-fold extension of T1 corresponding to seizure-free intervals of 218 days (longest ISI). The time to the 3rd GTC seizure day was 245 days in this dog. This dog had GTC seizures with rare focal onset with motor signs. LEV serum concentration was 11.6 mg/L in the dog with treatment success. Considering all dogs, the mean LEV serum concentration was 15.5 mg/L (5.5–52.2 mg/L; 10 dogs). Four dogs failed to achieve LEV serum concentrations within the human therapeutic range (10–40 mg/L; MVZ Labor Krone GmbH; Germany). Serum concentrations were unavailable from two other dogs, which died unexpectedly due to status epilepticus. There was no correlation between LEV serum concentration and extension of the interseizure interval (*p* = 0.173). Duration of postictal signs was negatively correlated with extension of the interseizure interval (*p* = 0.027), and KBr serum concentration was positively correlated with time to the 3rd GTC seizure day (0.05). Owners of seven dogs (58.3%; 7/12) chose to leave the study before 56 days of treatment due to a lack of perceived short-term efficacy of the treatment strategy. Five dogs adhered to the study protocol for ≥56 days. From these, two dogs experienced a ≥50% decrease in MSF compared to baseline in the first 56 days (2/12; 16.7%), but after 6 months of treatment, a decrease in MSF was only maintained in one dog (8.3%; 1/12). The 6 months drug retention rate was 25% (3/12) in the LEV group.

Effect on cluster seizures and focal seizures: cluster seizures stopped for ≥6 months in the three dogs with treatment success (11.5%; 3/26). Cluster seizures were the reason for study exit after the 3rd GTC day (early individual study endpoint) in two dogs from the PGB group and four dogs from the LEV group. Owners reported that twitches and jerks resembling focal motor seizures disappeared in three dogs from the PGB group and three dogs from the LEV group. One dog from the PGB group and two dogs from the LEV group newly developed signs resembling focal motor seizures.

### Tolerability

Side effects of the add-on treatment were more frequently reported when PGB was added to the treatment regimen. For the PGB group, mild-to-moderate increase in sedation (seven dogs), weakness (seven dogs), ataxia (six dogs), disorientation scores (five dogs), increased water uptake (three dogs), and flatulence (one dog) were reported. For the LEV group, an increase in ataxia (three dogs), disorientation (two dogs), restlessness scores (two dogs), vomiting (one dog), and flatulence (one dog) were reported. Dogs in the PGB group showed a significant increase in sedation score compared to baseline (*p* = 0.011) and to dogs in the LEV group (*p* = 0.041) (Supplementary Table S2). If necessary, side effects were managed with a stepwise 25% dosage decrease of PGB (two dogs, PGB group) or one baseline drug (three dogs, PGB group; two dogs, LEV group). No severe adverse events occurred and no relevant changes in laboratory parameters were observed.

### Follow-Up

Five dogs, which had failed to respond to PGB BID, entered treatment with PGB TID for 37–92 days (range; mean 68 days). Three of these five dogs required a subsequent decrease in PGB dose due to weakness and sedation. Yet, the mean PGB serum concentration increased from 2.9 to 4.6 mg/L in four dogs (unavailable from 1 dog). From the LEV group, two dogs entered treatment with a further increase in LEV dose by another 25% for 27 and 94 days, respectively. No further relevant extension of the interseizure interval or decrease in MSF compared to baseline was observed in either group.

Follow-up information was available for all dogs (Supplementary Table S1). PGB group: one of the two dogs with treatment success maintained a sustained decrease in seizures for 10 months without any other changes in ASMs or diet, but then relapsed with monthly single seizures and later on also cluster seizures. The other dog experienced 6 months of sustained seizure freedom, but then had a sudden drastic increase in seizure frequency with weekly seizures and was euthanized in status epilepticus 4 months later. One dog, which had not been treated with KBr yet due to lack of drug supply, was started on KBr despite a previous episode of pancreatitis, and PGB was tapered. This dog experienced one more episode of pancreatitis but thereafter remained seizure-free for 15 months until now. One dog became seizure-free after a change to a different commercial pet food diet without any further alteration of ASMs but then was euthanized 15 months after study completion due to a reason unrelated to epilepsy. One dog with ≥50% reduction in MSF in the first 56 days of PGB treatment died due to cluster seizures on day 60. LEV group: the dog with treatment success in the LEV group relapsed to monthly seizures after 218 seizure-free days, but with less severe and less frequent cluster seizures. One dog, which had not been treated with KBr yet due to lack of drug supply, was started on KBr despite a history of gastrointestinal disease and pancreatitis and thereafter experienced a seizure-free period of 6 months, then seizures recurred, but with ≥50% decrease in the frequency compared to study period. One dog with ≥50% reduction in MSF in the first 56 days died on the third seizure day (day 152).

In summary, at the time of writing, 10 dogs, seven dogs from the PGB group, and three dogs from the LEV group knowingly died or were euthanized due to their epileptic seizures. Two dogs from the PGB group were euthanized due to reasons unrelated to epileptic seizures. One dog in the PGB group became seizure-free >12 months and one dog in the LEV group had a significant reduction in seizures after the start with KBr. Seizures continued in the other 12 dogs despite continued treatment with ASMs.

## Discussion

Drug-resistance is a serious problem in dogs with idiopathic epilepsy. In this prospective treatment trial, we compared two treatment strategies, PGB add-on treatment with 4 mg/kg BID and a 30% increase in LEV add-on treatment in dogs with drug-resistant epilepsy. The study population was dogs with idiopathic epilepsy and frequent monthly seizures despite adequate treatment with licensed ASMs. All dogs had a history of cluster seizures.

Both drugs have different mechanisms of action than licensed veterinary drugs, which all modulate GABAergic mechanisms. PGB's major mechanism of action is the modulation of excitatory neurotransmitter release *via* binding to the alpha-2-delta subunit of neuronal voltage-gated calcium channels (34–41). LEV binds to the synaptic vesicle protein SV2A also modulating neurotransmitter release (42, 43). In a previous pilot study, PGB resulted in a favorable response in drug-resistant canine epilepsy and was well-tolerated (21). Furthermore, pharmacokinetic data in dogs suggest that therapeutic drug concentrations may be achieved if given BID (22), a fact that could significantly enhance owner compliance. A dose increase in LEV add-on treatment was chosen since the IVETF and ACVIM consensus guidelines advise to increase the LEV dosage or application interval in case of treatment failure with concurrent treatment with PB (5, 23).

Overall success rates were low with both strategies in this drug-resistant population. Threefold extension of the interseizure interval and a seizure-free period ≥3 months occurred only in 14.3% (two dogs) and 8.7% (one dog) in the PGB and LEV groups, respectively. Treatment success lasted 6, 10 (PGB), and 8 months (LEV) in these dogs; thereafter, monthly seizures reoccurred while ASM treatment was not changed. In one dog from the PGB group, treatment success was questionable because bromide serum concentration increased considerably during the study after a change in diet, presumably due to decreased chloride content in the new diet. Tubular reabsorption of bromide competes with chloride; thus, a diet low in chloride may lead to an increase in bromide serum concentrations. This observation underlines the need for long-term follow-up and strict control of diet and drug concentrations when conducting clinical studies on drug-resistant epilepsy. Results and low-response rates could also be attributed to long-term fluctuations and the waxing and waning patterns of seizure occurrence in canine epilepsy (44, 45). Placebo rates as high as 30% were described in other studies previously and may also be relevant for the interpretation of head-to-head trials (44). Nevertheless, the present study protocol and outcome parameters, i.e., minimum of 3-fold extension of the longest baseline interseizure interval appear less sensitive to placebo and regression to the mean effects.

Comparing our results with those of Dewey et al. (21) is challenging due to the different types of analysis and primary outcome parameters. Dewey et al. (21) treated a similar cohort of dogs with idiopathic epilepsy, which was pharmaco-resistant to PB or KBr and had similar seizure frequencies (MSF 4.2 vs. 3.8 in this study), with PGB TID as an add-on medication. The authors reported 7/11 dogs (63.6%) with ≥50% decrease in MSF within the first 3 months of treatment and a mean reduction in MSF of 57% in nine dogs. Defining responders by a ≥50% decrease in seizure frequency may be more prone to variations and a placebo effect than the 3-fold extension of the interseizure interval [as shown in a previous study from our group investigating imepitoin in a placebo-controlled trial in head tremor, (46)]. It should be noted that long-term follow-up was not evaluated in the study by Dewey et al. (21), whereas our follow-up data showed that responder rates with ≥50% decrease in MSF rapidly declined. Furthermore, we failed to observe an additional positive effect on seizure frequency or interseizure intervals in five dogs that underwent dose escalation with PGB TID dosing.

There are several possibilities for the lack of efficacy of PGB BID application in this cohort: insufficient drug serum concentrations, restriction of the efficacy of PGB to focal-onset seizures, or changes in target structures and seizure propagating mechanisms in dogs with chronic epilepsy. PGB 4 mg/kg BID led to serum concentrations within the human therapeutic range in all but one dog. In this regard, it should be mentioned that previously reported mean PGB serum concentrations were higher (21) (6.8 mg/L) than in the present investigation with BID (3.8 mg/L) or TID (4.6 mg/L) application of respective dosages. A linear relationship between PGB dose and serum concentration, and PGB dose and treatment efficacy exists in humans for the treatment of focal-onset seizures (47–50). In line with these observations in human medicine, there was a trend toward a moderate positive correlation between PGB drug serum concentration and extension of the longest ISI in our cohort of dogs (rho = 0.515, *p* = 0.060).

Pregabalin is only licensed for adjunctive therapy of focal and focal-onset seizures evolving into bilateral tonic–clonic seizures in humans besides neuropathic pain and generalized anxiety disorder (51). In this aspect, anti-seizure efficacy for focal and focal-onset seizures has been well-documented with ≥50% seizure reduction in >40% of human patients (18, 49, 50, 52). Recent investigations in children and adults failed to demonstrate a significant effect of PGB on generalized-onset tonic–clonic seizures compared to placebo (53). In dogs with idiopathic epilepsy, the most frequent seizure type is “focal epileptic seizure” evolving into generalized epileptic seizures (27), which is the equivalent of “focal to bilateral tonic–clonic seizure” in humans (54). Classification of epilepsies by predominant seizure types in dogs analogous to human medicine faces specific challenges, relying on owner observations, difficulties to recognize non-motor signs at seizure onset, and dependence on the interpretation of the investigator without routine support by electroencephalography. Focal epileptic seizure onset was suspected in 46.2% (12/26; six PGB; six LEV) of dogs in the study and was the predominant seizure type in 26.9% (7/26; 4 PGB; 3 LEV). The two dogs with treatment success in the PGB group had suspected focal-onset seizures (one rare motor, one frequent autonomic).

Treatment with increased dosages of LEV add-on treatment is a popular treatment strategy in canine drug-resistant epilepsy and aims at overcoming tolerance issues. In our study, only one dog showed treatment success indicating poor efficacy of this treatment strategy in our cohort of dogs. However, it should be considered that the recruitment process of our study may have already been selected for LEV non-responders. Thus, treatment failure could result from genetic or molecular factors of these dogs' epilepsy not being responsive to LEV's mechanism of action (55). Alternatively, a honeymoon effect may have occurred, i.e., a decrease or loss of efficacy of LEV when used chronically as previously described (55–58). Decrease or lack of efficacy could be related to functional tolerance issues, i.e., pharmacodynamic tolerance due to reduced effects at target structures, or pharmacokinetic tolerance due to increased metabolism of LEV, especially when used as an add-on to PB. The 30% dosage increase of LEV failed to achieve drug concentrations within the human therapeutic range in four dogs in this study Therefore, the LEV dosage increase of 30% in our study might have been insufficient to achieve further treatment effect. In this context, it should be noted that serum concentrations as a guide for LEV add-on treatment are still controversial due to an equivocal relationship between efficacy and drug serum concentrations in humans (23, 59, 60). So far, there is no established therapeutic range for LEV serum concentrations in dogs. Nevertheless, there are concerns that LEV serum concentrations might decrease and be too low with time and when concurrent treatment with PB is applied (24). Measurement of LEV serum concentrations could at least ascertain that drug concentrations within the human therapeutic range are achieved (5, 23). A strategy with continued add-on treatment with LEV at increased dosages may be inappropriate to overcome tolerance issues. Considering previously published results (57), LEV pulse therapy might be the preferred therapeutic strategy for dogs with cluster seizures to address drug tolerance issues and avoid a honeymoon effect (57).

The study protocol allowed for an individual early study end if no relevant short-term success was obtained, with the endpoint being the 3rd GTC seizure day during the treatment phase. This avoided prolonged adherence to a presumably ineffective treatment protocol and aimed at increasing owner compliance. It may be of interest that most dogs remained in the study beyond this early individual endpoint. But finally, five dogs in the PGB and seven dogs in the LEV group exited the study before 56 days of treatment due to persistent GTC seizures. For future studies, a later individual study endpoint, e.g., time to the 4th or 5th seizure day could be discussed (4, 31, 32). Since the current study protocol was unintendedly selected for short-term treatment success, disease modifying effects might have been missed, and the antiseizure effects of these therapeutic strategies were underestimated. On the other hand, short-term treatment success correlated with long-term treatment success in the case of imepitoin (8). In general, longer treatment periods with a supposedly ineffective ASM may also result in higher drop-out rates or lower owner compliance including failure to record seizures precisely and thus contributing to a placebo effect from the inclusion of pseudo-responders. Furthermore, the inclusion of all dogs that entered the treatment protocol into the final analysis (an intention-to-treat analysis) avoids reporting false high efficacy rates, which may occur if only dogs improving during the therapeutic intervention are analyzed. We, therefore, suggest that our results and this treatment protocol reflect a real-life situation.

The dogs in this study represent canine patients in veterinary practice with a need for treatment strategies beyond the drugs licensed for use in dogs with idiopathic epilepsy (9). In humans, older studies reported a chance of ≥50% decrease in seizure frequency in 19–29% of patients after two previously failed ASM trials (61, 62). Newer studies applying the current International League Against Epilepsy definitions of seizure freedom report that 4.4–27% may become seizure-free with the 3rd ASM (28, 63–66). The short-term response rates in our study are in line with these assumptions; however, observations on long-term outcomes revealed recurrence of monthly seizures after 6–10 months.

There were multiple limitations to the study. Only 50% of the dogs had diagnostic imaging of the brain performed; thus, subtle structural brain lesions contributing to drug-resistance may have been overlooked. Dogs neither were primarily randomized to the study groups nor were the investigators blinded. However, in randomized trials, a very high number of participants is warranted to assure equal groups, which is addressed by the matched pair design of the study.

The strict inclusion criteria selected for a rather chronic, difficult-to-treat group of dogs with a high seizure burden. This is also reflected in the fact that all participating dogs suffered from cluster seizures although this was not an inclusion criterion. It remains undefined whether the response rates of the study would have been higher in a less severely affected population of dogs with idiopathic epilepsy.

The long baseline period of 4 months aimed to compensate for variations in seizure frequencies; however, it is still possible that dogs were enrolled at a state of disease progression or natural fluctuation of disease (4, 44, 45). Furthermore, in the PGB group, side effects made drug adaptions necessary in a considerable proportion of dogs (35.7%; three baseline drugs, two PGB dose, Supplementary Table S1). These adaptions of the baseline ASMs reflect daily clinical practice but may have led to some decrease in the antiseizure efficacy of the baseline drug, which had to be compensated by the PGB add-on therapy.

In conclusion, this study design with an early individual study endpoint was associated with high compliance of dog owners and enabled analysis of all study participants. The overall low success rates with both treatment strategies likely represent a real-life situation in canine drug-resistant idiopathic epilepsy in daily veterinary practice. The occurrence of epilepsy-related deaths, even in dogs with a favorable response, prompts the need for investigation of better pharmacologic and non-pharmacologic treatment strategies in dogs with drug-resistant idiopathic epilepsy. Future studies in PGB treatment may imply dose escalations guided by drug serum concentrations.

## Data Availability Statement

Theoriginal contributions presented in the study are included in the article/Supplementary Material, further inquiries can be directed to the corresponding author/s.

## Ethics Statement

The animal study was reviewed and approved by Ethics Committee (AZ 168-02-05-2019) of the veterinary faculty of the Ludwig-Maximilians-Universität Munichand conducted in accordance with the German Animal Welfare Act. Written informed consent was obtained from the owners for the participation of their animals in this study.

## Author Contributions

AF, FW, KJ, and SK designed the study. SK conducted the experiments and wrote the first draft of the manuscript. TS, GB, SD, HS, and KJ contributed cases and collected data. AF and KJ supervised case collections. SR, AF, and HP provided input into statistics. AF, KJ, and HP reviewed the first draft of the manuscript. All authors provided input into the final version of the manuscript.

## Funding

This study was funded by AniCura, 182 32Danderyd, Sweden, as part of a research fund project from 2019 to 2021. AniCura is an affiliate of Mars Incorporated.

## Conflict of Interest

The authors declare that the research was conducted in the absence of any commercial or financial relationships that could be construed as a potential conflict of interest.

## Publisher's Note

All claims expressed in this article are solely those of the authors and do not necessarily represent those of their affiliated organizations, or those of the publisher, the editors and the reviewers. Any product that may be evaluated in this article, or claim that may be made by its manufacturer, is not guaranteed or endorsed by the publisher.

## Abbreviations

PB: phenobarbital;KBr potassium bromide
ASM: antiseizure medication
BID: every 12 h
TID: every 8 h
ACVIM: American College of Veterinary Internal Medicine
IVETF: International Veterinary Epilepsy Task Force
GTC: generalized tonic–clonic
MRI: magnetic resonance imaging
CSF: cerebrospinal fluid
T1: longest baseline interseizure interval
MSF: monthly seizure frequency
MSDF: monthly seizure day frequency
MCDF: monthly cluster seizure day frequency
longest ISI: longest interseizure interval during treatment phase
SD: standard deviation
PGB: pregabalin
LEV: levetiracetam.
