# Supplementary material for: Pregabalin Add-On vs. Dose Increase in Levetiracetam Add-On Treatment: A Real-Life Trial in Dogs With Drug-Resistant Epilepsy
Source: Front Vet Sci. 2022 Jul 6;9:910038. doi: 10.3389/fvets.2022.910038 (PMC9298511; doi:10.3389/fvets.2022.910038)
Supplement: Supplementary file 1 [file Data_Sheet_1.pdf]

Table S1: Study participants and outcome

dogs	group	match	breed	sex	age of onset (y)	age at inclusion (y)	duration of epilepsy (y)	seizure type	focal seizure onset	MRI, CT, CSF, EEG workup	baseline ASMs	previous ASMs (failed)	previous ASMs (non-tolerated)
1	PGB	24	Australian Shepherd	m	3.33	6.25	3.08	GTC	frequent, motor left		PB, KBr		increase in KBr
2	PGB	23	Retriever Mix	m	1.33	3.17	2.08	GTC	no	MRI, CSF	PB, LEV, amantadine		KBr
3	PGB		Australian Shepherd	mn	1.50	9.58	7.25	GTC	no		PB, LEV	IMP, KBr	
4	PGB	19	Golden Retriever	m	1.08	3.50	1.75	GTC	rare, motor bilateral	EEG	PB, KBr, LEV	IMP	
5	PGB	21	Australian-Shepherd/Border Collie mix	mn	4.42	6.92	2.5	GTC	no		PB	IMP	KBr not available*
6	PGB	26	Great Dane Mix	fs	1.50	3.50	2.17	GTC	no		PB, KBr		
7	PGB	22	Beagle	f	2.25	2.75	0.58	GTC	frequent, motor bilateral	MRI	PB, KBr		
8	PGB	20	Border Terrier	fs	4.75	8.33	3.58	GTC		MRI, CSF, EEG	PB, KBr, LEV	IMP	
9	PGB	17	White German Shepherd	m	2.75	3.58	2.83	GTC		MRI, CSF	PB, KBr, LEV	IMP	
10	PGB	16	Boxer	f	0.67	2.50	1.83	GTC	rare, motor left	CT, CSF	PB, KBr		
11	PGB	25	Schafpudel	f	3.17	9.42	6.33	GTC	no		PB, KBr		
12	PGB		Cane Corso Italiano	m	0.92	1.92	1	GTC	frequent, autonomic	CT	PB, KBr	IMP, LEV	
13	PGB	18	Mixed breed	mn	1.67	2.67	0.83	GTC	frequent, autonomic		PB, KBr		
14	PGB	15	Old English Bulldog	m	1.00	2.75	1.67	GTC	no		PB, KBr	IMP	
15	LEV	14	Australian Shepherd/ Cocker Spaniel mix	fs	0.92	4.25	3.58	GTC	rare, motor right	MRI, CSF, EEG	PB, LEV, PGB	topiramate	KBr
16	LEV	10	Golden Retriever	m	1.67	4.08	2.42	GTC	frequent, motor bilateral		PB, KBr, LEV, PGB		
17	LEV	9	Labrador	mn	2.58	6.58	4	GTC/GC	frequent, motor uni- and bilateral	MRI, CSF, EEG	PB, LEV	IMP, KBr	
18	LEV	13	Mixed breed	fs	1.42	6.25	4.83	GTC	rare, autonomic	MRI, CSF	PB, KBr, LEV, PGB	IMP, gabapentin	
19	LEV	4	Magyar-Viszla	m	1.08	3.00	1.92	GTC			PB, KBr, LEV	IMP	
20	LEV	8	Mixed breed	m	4.00	9.08	5.08	GTC	no	MRI, CSF	PB, KBr, LEV, topiramate	IMP	
21	LEV	5	Mixed breed	mn	2.67	6.00	3.5	GTC	no		PB, LEV		KBr not available*
22	LEV	7	Elo	m	4.58	5.58	1.17	GTC		MRI, CSF	LEV	PB	KBr not available*
23	LEV	2	Siberian Husky	m	1.08	4.00	2.92	GTC	rare, motor bilateral		PB, LEV	KBr	
24	LEV	1	Old German Shepherd Dog	m	3.50	5.58	1.08	GTC		CT, CSF	LEV, gabapentin		PB; KBr not available*
25	LEV	11	Rhodesian Ridgeback	fs	3.75	7.75	4.08	GTC	frequent, motor right	MRI, CSF	PB, KBr, LEV, PGB	IMP	increase in KBr
26	LEV	6	Mixed breed	mn	3.00	11.33	8	GTC	no	MRI	PB, KBr, LEV		IMP

*due to lack of drug supply

Table S1:

dogs	group	study drug baseline (mg/kg)	study drug study (mg/kg)	PB conc baseline (mg/l)	PB conc study (mg/l)	KBr conc baseline (mg/l)	KBr conc study (mg/l)	PGB conc BID (mg/l)	LEV conc baseline (mg/l)	LEV conc study (mg/l)	T1 (days)	longest ISI (days)	change ISI (long. ISI/T1)	MSF baseline	MSF study (56 days)
1	PGB		4.08	26.4	22.5	1624	<300**	7.80			24	30	1.25	2.50	1.50
2	PGB		3.95	30	28.7			2.96	41.20	20.30	15	9	0.60	5.25	
3	PGB		4.03	22.2	23.2			3.90	2.30	8.60	32	36	1.13	4.75	2.00
4	PGB		3.66	24.4	22	1841	2044	3.21	2.40	20.50	31	6	0.19	4.25	
5	PGB		3.87	30	27.1			4.20			28	59	2.11	2.30	1.50
6	PGB		4.02	25.3	25.3	2016	2195	5.90			38	51	1.34	1.50	1.50
7	PGB		3.91	24.5	20.6	1007	1032	1.40			19	14	0.74	5.75	
8	PGB		4.08	26.3	31.3	1949	1788	2.20	0.00	4.00	17	40	2.35	3.00	1.00
9	PGB		4.10	24.9	21.2	867	821	2.60	4.90	3.30	10	7	0.70	7.50	
10	PGB		4.00	37.5	38.6	1075	1779	6.30			28	180	6.43	2.00	0.00
11	PGB		3.98	29.6	25.5	1906	2040	2.40			37	29	0.78	1.25	1.00
12	PGB		3.86	34.6	30.7	1191	915	3.80			30	28	0.93	5.00	2.50
13	PGB		3.95	21.9	24.4	1450	1367	4.10			37	122	3.30	1.50	0.00
14	PGB		4.00	23.3	22.1	1616	1430	2.60			23	16	0.70	9.75	12.50
15	LEV	19.70	25.00	27.4	22.1				14.40	11.60	25	218	8.72	7.00	0.50
16	LEV	17.20	22.99	24.4	26.7	1059	933		2.20	4.90	30	12	0.40	1.50	
17	LEV	21.40	27.78	36	30.4				11.00	13.80	6	8	1.33	9.50	9.00
18	LEV	32.60	43.48	23.9	25	1855	3130		5.50	7.20	18	21	1.17	1.75	1.50
19	LEV	20.00	25.00	32.9		994			0.00		36	19	0.53	4.00	
20	LEV	20.83	27.08	18.2	20.1	1742	2264		8.50	8.20	15	15	1.00	2.75	
21	LEV	23.00	28.80	26.3					11.10		36	74	2.06	7.50	0.00
22	LEV	31.00	38.82						20.80	13.90	24	9	0.38	3.50	
23	LEV	37.75	50.34	28.2	33.5				10.50	20.10	20	13	0.65	3.00	
24	LEV	20.10	26.88						27.60	52.20	30	9	0.30	2.25	
25	LEV	19.70	26.32	21.4	22.2	1585	1358		7.60	5.50	27	27	1.00	1.75	2.00
26	LEV	22.70	30.30	19.8	18.8	1662	1681		7.50	17.70	39	15	0.38	2.50	

**discontinued due to side effects (weakness, vomiting)

Table S1:

dogs	group	change MSF	MSDF baseline	MSDF study (56 days)	change MSDF	MCDF baseline	MCDF study (56 days)	change MCDF	no clusters for ≥ 6 mo	focal sz. baseline	focal sz. study	time to 3rd seizure day (d)	study exit after *x* seizure days	study duration (d)	dose reduction due to side effects
1	PGB	-40.00%	1.75	1.00	-42.86%	1.25	0.5	-60.00%	no	no	no	58	4	75	baseline ASM
2	PGB		4.25			1.00			no	yes	yes	22	3	26	
3	PGB	-57.89%	4.25	1.00	-76.47%	2.25	1.0	-55.56%	no	yes	no	67	5	84	PGB
4	PGB		2.50			1.50			no	yes	yes	9	3	9	
5	PGB	-34.64%	1.61	0.50	-68.88%	0.69	0.5	-27.38%	no	no	no	101	4	151	
6	PGB	0.00%	1.00	0.50	-50.00%	0.50	0.5	0.00%	no	yes	no	110	7	168	baseline ASM
7	PGB		3.00			1.75			no	yes	yes	30	4	45	PGB
8	PGB	-66.67%	2.75	1.00	-63.64%	0.25	0.5	100.00%	no	yes	yes	79	4	103	
9	PGB		6.00			1.00			no	yes	yes	13	11	38	
10	PGB	-100.00%	1.25	0.00	-100.00%	0.75	0.0	-100.00%	yes	no	no	219	3	219	baseline ASM
11	PGB	-20.00%	1.25	1.00	-20.00%	0.25	0.0	-100.00%	no	no	no	66	5	116	
12	PGB	-50.00%	2.25	1.50	-33.33%	1.00	1.0	0.00%	no	no	yes	49	4	60	
13	PGB	-100.00%	1.50	0.00	-100.00%	0.25	0.0	-100.00%	yes	no	no	205	3	205	
14	PGB	28.21%	3.50	4.50	28.57%	3.50	4.5	28.57%	no	yes	no	22	8	59	
15	LEV	-92.86%	3.00	0.50	-83.33%	2.25	0.0	-100.00%	yes	yes	no	245	3	245	
16	LEV		1.00			1.75			no	yes	no	24	5	47	
17	LEV	-5.26%	9.25	8.50	-8.11%	0.75	0.5	-33.33%	no	no	no	8	18	92	
18	LEV	-14.29%	1.75	1.50	-14.29%	0.00	0.0	0.00%	no	yes	no	50	4	88	
19	LEV		2.50			1.75			no	yes	yes	21	5	23	
20	LEV		2.25			0.25			no	no	no	36	3	50	baseline ASM
21	LEV	-100.00%	1.25	0.00	-100.00%	1.00	0.0	-100.00%	no	no	yes	152	3	152	
22	LEV		2.50			1.00			no	yes	yes	10	5	22	
23	LEV		2.50			0.00			no	yes	yes	19	3	35	
24	LEV		1.50			0.25			no	no	yes	15	4	28	
25	LEV	14.29%	1.50	2.00	33.33%	0.50	1.0	100.00%	no	yes	yes	41	6	64	baseline ASM
26	LEV		1.00			1.00			no	yes	yes	36	3	38	

Table S1:

dogs	group	drug retention 6 mo after study inculsion***	follow-up
1	PGB	yes	seizures every 4-6 weeks
2	PGB	no	died in status epilepticus
3	PGB	yes	euthanasia not related to epilepsy 14 months after study completion
4	PGB	no	approx. 2 seizures per month
5	PGB	no	seizure-free after start of KBr
6	PGB	yes	euthanized due to intestinal foreign body probably due to drug-related polyphagia
7	PGB	no	euthanized in status epilepticus
8	PGB	yes	approx. 2 seizures per month
9	PGB	no	euthanized due to uncontrolled seizures
10	PGB	yes	died in status epilepticus
11	PGB	no	died in status epilepticus
12	PGB	no	euthanized in status epilepticus
13	PGB	yes	recurrence of monthly seizures after 2 seizures in 10 months
14	PGB	no	cluster seizures every 6 weeks
15	LEV	yes	recurrence of monthly, but less severe seizures after 7 months seizure freedom
16	LEV	no	seizures every 2 weeks
17	LEV	no	daily seizures
18	LEV	no	seizures every 4-8 weeks
19	LEV	no	euthanized in status epilepticus
20	LEV	yes	seizures every 2-4 weeks
21	LEV	no	died in status epilepticus
22	LEV	no	≥ 50% decrease in seizure frequency after start of KBr
23	LEV	no	seizures every 9-18 days
24	LEV	no	euthanized due to cluster seizures
25	LEV	yes	seizures every 2-8 weeks
26	LEV	no	one seizure-free interval of 11 weeks, then seizures every 2-4 weeks

***dogs that died during the study were allocated to "no"

legend	
m/mn	male/male neutered
f/fs	female/female spayed
y	years
d	days
GTC/GC	generalized tonic-clonic/generalized clonic
ASM	antiseizure medication
conc	serum concentration
PGB	pregabalin
LEV	levetiracetam
PB	phenobarbital
KBr	potassium bromide
IMP	imepitoin
BID	dosing q12 hours po
TID	dosing q8 hours po
T1	longest interseizure interval during 4 months baseline
long. ISI	longest interseizure interval during treatment phase
MSF	monthly seizure frequency
MSDF	monthly seizure day frequency
MCDF	monthl cluster day frequency
focal sz.	focal seizures

Table S1:

**dogs**	**group**
1	PGB
2	PGB
3	PGB
4	PGB
5	PGB
6	PGB
7	PGB
8	PGB
9	PGB
10	PGB
11	PGB
12	PGB
13	PGB
14	PGB
15	LEV
16	LEV
17	LEV
18	LEV
19	LEV
20	LEV
21	LEV
22	LEV
23	LEV
24	LEV
25	LEV
26	LEV
