# Supplementary material for: Pregabalin Add-On vs. Dose Increase in Levetiracetam Add-On Treatment: A Real-Life Trial in Dogs With Drug-Resistant Epilepsy
Source: Front Vet Sci. 2022 Jul 6;9:910038. doi: 10.3389/fvets.2022.910038 (PMC9298511; doi:10.3389/fvets.2022.910038)
Supplement: Supplementary file 2 [file Data_Sheet_2.PDF]

Table S2: Behavioural characteristics and owner assessment scores

Parameter	Baseline			Treatment phase			Mean % change treatment vs. baseline score		
	**Pregabalin n = 14**	**Levetiracetam n = 12**	***p-value***	**Pregabalin n = 14**	**Levetiracetam n = 12**	***p value***	**Pregabalin n = 14**	**Levetiracetam n = 12**	***p value***
Playfulness, mean (range)*	5.3 (0-10)	6.0 (2-10)	*0.634*	6.3 (1-10)	6.0 (2-10)	*0.836*	+19.7%	+1.0%	*0.174*
Activity level, mean (range)*	6.2 (2-9)	6.4 (2-10)	*0.842*	6.4 (2-9)	6.3 (2-10)	*0.982*	+2.9%	-1.3%	*0.349*
**Side effect scores#**									
Weakness, mean (range)	2.8 (0-6)	4.4 (0-10)	*0.202*	4.2 (0-8)	4.5 (0-10)	*0.803*	+50.0%	+2.8%	*0.699*
Ataxia, mean (range)	2.3 (0-6)	4.1 (0-10)	*0.137*	3.3 (0-8)	4.5 (0-10)	*0.339*	+44.4%	+9.6%	*0.222*
Disorientation, mean (range)	1.3 (0-6)	2.0 (0-4.5)	*0.715*	1.6 (0-5)	2.3 (0-6)	*0.461*	+28.6%	+18.5%	*0.905*
Sedation, mean (range)	0.9 (0-5)	4.3 (0.5-8)	*0.088*	2.3 (0-8)	4.1 (0-8)	*0.187*	+160.0%	-4.9%	***0.041***
Restlessness, mean (range)	2.6 (0-7)	1.2 (0-6)	*0.160*	2.5 (0-8)	1.5 (0-7)	*0.287*	-5.4%	+25.0%	*0.704*
Increased appetite, mean (range)	7.7 (0-10)	9.3 (2.5-10)	*0.055*	8.2 (1-10)	9.4 (2-10)	*0.082*	+6.5%	+0.9%	*0.157*
Sum of side effect scores	17.5	25.4	*0.667*	22.1	26.3	*0.815*			

Significant values ($p < 0.05$) are bolded.
*scores: 0 – 10; 0, very low; 10, excellent
#scores: 0 – 10; 0, none; 10, severe
